# Antibodies to Enteroviruses in Cerebrospinal Fluid of Patients with Acute Flaccid Myelitis

**DOI:** 10.1128/mBio.01903-19

**Published:** 2019-08-13

**Authors:** Nischay Mishra, Terry Fei Fan Ng, Rachel L. Marine, Komal Jain, James Ng, Riddhi Thakkar, Adrian Caciula, Adam Price, Joel A. Garcia, Jane C. Burns, Kiran T. Thakur, Kimbell L. Hetzler, Janell A. Routh, Jennifer L. Konopka-Anstadt, W. Allan Nix, Rafal Tokarz, Thomas Briese, M. Steven Oberste, W. Ian Lipkin

**Affiliations:** a Center for Infection and Immunity, Mailman School of Public Health, Columbia University, New York, New York, USA; b Division of Viral Diseases, Centers for Disease Control and Prevention, Atlanta, Georgia, USA; c Department of Pediatrics, University of California San Diego School of Medicine, La Jolla, California, USA; d Division of Critical Care and Hospitalist Neurology, Department of Neurology, Columbia Irving University Medical Center, New York, New York, USA; e Division of Viral Diseases, Centers for Disease Control and Prevention, Atlanta, Georgia, USA; Brown University; University of Alabama at Birmingham; National Institutes of Health

**Keywords:** VirCapSeq-VERT, acute flaccid myelitis, antibodies, enterovirus, enterovirus D-68, peptide array, serology

## Abstract

Acute flaccid myelitis (AFM) has caused motor paralysis in >560 children in the United States since 2014. The temporal association of enterovirus (EV) outbreaks with increases in AFM cases and reports of fever, respiratory, or gastrointestinal illness prior to AFM in >90% of cases suggest a role for infectious agents. Cerebrospinal fluid (CSF) from 14 AFM and 5 non-AFM patients with central nervous system (CNS) diseases in 2018 were investigated by viral-capture high-throughput sequencing (VirCapSeq-VERT system). These CSF and serum samples, as well as multiple controls, were tested for antibodies to human EVs using peptide microarrays. EV RNA was confirmed in CSF from only 1 adult AFM case and 1 non-AFM case. In contrast, antibodies to EV peptides were present in CSF of 11 of 14 AFM patients (79%), significantly higher than controls, including non-AFM patients (1/5 [20%]), children with Kawasaki disease (0/10), and adults with non-AFM CNS diseases (2/11 [18%]) (*P* = 0.023, 0.0001, and 0.0028, respectively). Six of 14 CSF samples (43%) and 8 of 11 sera (73%) from AFM patients were immunoreactive to an EV-D68-specific peptide, whereas the three control groups were not immunoreactive in either CSF (0/5, 0/10, and 0/11; *P* = 0.008, 0.0003, and 0.035, respectively) or sera (0/2, 0/8, and 0/5; *P* = 0.139, 0.002, and 0.009, respectively).

## Abstract (precis)

The presence in cerebrospinal fluid of antibodies to EV peptides at higher levels than non-AFM controls supports the plausibility of a link between EV infection and AFM that warrants further investigation and has the potential to lead to strategies for diagnosis and prevention of disease.

## INTRODUCTION

Neurotropic enteroviruses (EVs), such as poliovirus, have long been associated with paralytic disease. While there has been a pronounced decrease in poliomyelitis cases worldwide due to global polio eradication efforts (1), a similar paralytic syndrome, acute flaccid myelitis (AFM), has raised concerns that another EV may be implicated (2). AFM presents with acute flaccid weakness in one or more limbs with depressed tendon reflexes. Some patients have cranial nerve abnormalities, including facial weakness, dysarthria, or dysphagia. The majority of affected individuals are children (3). Most patients report a respiratory, or gastrointestinal illness in the 4 weeks preceding disease onset. Cases occur in the United States with an every-other-year periodicity during the late summer and early fall months (www.cdc.gov/acute-flaccid-myelitis/afm-cases.html).

The cause of AFM remains elusive. An infectious agent is only rarely detected in cerebrospinal fluid (CSF). In the initial CDC investigation, the presence of EV RNA by reverse transcription-PCR (RT-PCR) was demonstrated in the CSF from only 1 of 55 children with confirmed AFM. However, more than 40% of children with AFM had PCR evidence of EV RNA in respiratory or fecal samples, suggesting a possible role for EV in AFM (4). Failure to detect viral RNA in CSF by PCR may represent absence of virus, low levels of viral template, or sequence mismatch between viral template and primers or probes. Unbiased high-throughput sequencing can address the challenge of sequence mismatch; however, it is less sensitive than real-time PCR because abundant host RNA competes with viral RNA in sequencing reactions. Another alternative is the VirCapSeq-VERT, a positive-selection high-throughput sequencing system that detects viral genetic material with sensitivity equivalent to real-time PCR (5). A third alternative is to focus on immunological responses to pathogens that may not be present in the materials sampled (6, 7). In this study, we used both viral-capture high-throughput sequencing (VirCapSeq-VERT system) and peptide microarray approaches to examine the role of EV in AFM, using specimens collected from AFM patients and three control groups of patients with either non-AFM central nervous system (CNS) or inflammatory illnesses.

## RESULTS

Samples from 14 confirmed AFM patients were analyzed (Table 1). With the exception of one adult, all were pediatric patients with a median age of 3 years (range, 1 to 7 years); 69% were male. The median age for the AFM group was 3 years, compared to 11 years for patients in the non-AFM CNS disease control group (NAC), 0.5 year for patients in the Kawasaki disease control group (KDC group), and 46 years for adults with non-AFM CNS diseases (adult control [AC] group) (*P* = 0.0098, <0.0001, and <0.0001, respectively) (Table 1; see Text S1 in the supplemental material). The 14 AFM and 5 NAC specimens from 2018 were collected in a similar period (AFM, weeks 8 to 38; NAC, weeks 26 to 37; *P* = 0.6317, Mann-Whitney test) (Text S1). Whereas KDC specimens were collected between 2005 and 2019, AC specimens were collected in 2018.

**TABLE 1:** Baseline study characteristics of the patient samples^*a*^

Study group	No. of samples		Sex (% male)	Age range (median)	Collection yr	Sample no.	CSF	Serum	Note(s)	EV PCR/typing result		
										Detected pathogen		Suspected pathogen (stool)
	CSF	Paired sera								CSF^*b*^	Respiratory specimen	
AFM	13	10	69	1–7 yr (3 yr)	2018	AFM_1	Y	Y		Neg	EV-D68	Neg
						AFM_2	Y	Y		Neg	EV-D68	NA
						AFM_4	Y	N		Neg	NA	NA
						AFM_5	Y	Y		Neg	Neg	NA
						AFM_6	Y	N		Neg	NA	NA
						AFM_8	Y	Y		Neg	Neg	Neg
						AFM_12	Y	Y		Neg	Neg	CVA4
						AFM_13	Y	N		Neg	NA	Neg
						AFM_14	Y	Y		Neg	Neg	CVA9
						AFM_17	Y	Y		Neg	EV-D68	Neg
						AFM_18	Y	Y		Neg	NA	NA
						AFM_19	Y	Y		Neg	RV-A81	Neg
						AFM_20	Y	Y		Neg	Neg	NA
	1	1	Female	Adult	2018	AFM_7	Y	Y		EV-A71	Neg	EV-A71

NAC	5	2	60	0.8–81 yr (11 yr)	2018	Non_AFM_3	Y	Y	Cause unknown	Neg	NA	Neg
						Non_AFM_9	Y	N	West Nile case	Neg	NA	NA
						Non_AFM_10	Y	N	Transverse myelitis	Neg	NA	E-9
						Non_AFM_11	Y	Y	Longitudinally extensive myelitis	Neg	Neg	Neg
						Non_AFM_16	Y	N		E-25	E-25	E-25
	1	0	Female	15	2019	AC-KT-076	Y	N	Autoimmune encephalitis	NA	NA	NA

KDC	10	8	55	2–36 mo (6.8 mo)	2006	KD_824	Y	Y		NA	NA	NA
					1999	KD_98	Y	N		NA	NA	NA
					2007	KD_3340	Y	Y		NA	NA	NA
					2008	KD_3424	Y	Y		NA	NA	NA
					2018	KD_3589	Y	Y		NA	NA	NA
					2018	KD_183068	Y	Y		NA	NA	NA
					2018	KD_183041	Y	Y		NA	NA	NA
					2019	KD_193017	Y	Y		NA	NA	NA
					2008	KD_3355	Y	N		NA	NA	NA
					2005	KD_3181	Y	Y		NA	NA	NA

AC	11	5	45	21–73 yr (45.8 yr)	2018	AC-KT-010	Y	N	Viral meningitis, unspecified (A87.9)	NA	NA	NA
					2018	AC-KT-012	Y	Y	Possible viral meningitis	NA	NA	NA
					2018	AC-KT-017	Y	Y	Systemic lupus erythematosus	NA	NA	NA
					2018	AC-KT-021	Y	N	Cryptococcal meningitis	NA	NA	NA
					2018	AC-KT-023	Y	Y	PML	NA	NA	NA
					2018	AC-KT-024	Y	N	Myalgia, CA of prostate, squamous cell carcinoma lung, neoplasia following lung transplant, muscle pain likely secondary to cetuximab infusion	NA	NA	NA
					2018	AC-KT-025	Y	Y	Unspecified epilepsy	NA	NA	NA
					2018	AC-KT-029	Y	Y	Stiff person syndrome	NA	NA	NA
					2018	AC-KT-030	Y	N	Multiple sclerosis	NA	NA	NA
					2018	AC-KT-042	Y	N	Multiple sclerosis	NA	NA	NA
					2018	AC-KT-049	Y	Y	Demyelinating disease—likely multiple sclerosis	NA	NA	NA

a All samples were deidentified and met criteria for nonhuman subject research at Columbia University and the University of California, San Diego. For samples contributed by CDC, the work was determined by the CDC National Center for Immunization and Respiratory Diseases to constitute public health surveillance rather than human subject research. Y, yes; N, no; Neg, negative; NA, not available; PML, progressive multifocal leukoencephalopathy.

b Combined results of PCR-based EV testing performed by the CDC and CII of Columbia University.

10.1128/mBio.01903-19.1 TEXT S1 Supplemental appendix showing AFM and non-AFM control (NAC) sample characteristics and comparisons. Download Text S1, DOCX file, 0.1 MB. Copyright © 2019 Mishra et al. 2019 Mishra et al. This content is distributed under the terms of the Creative Commons Attribution 4.0 International license.

### Virologic testing and VirCapSeq-VERT.

Virologic testing was performed on all CSF samples from AFM and NAC cases, as well as fecal and respiratory specimens (Table 1). EV-A71 was identified in CSF and feces of 1 of 14 (7%) AFM cases. One of 5 (20%) non-AFM cases was positive for echovirus 25 (E-25) in CSF, respiratory, and fecal samples (Table 1). Seven of 14 (50%) AFM cases and 2 of 6 (33%) non-AFM cases were positive for EV in corresponding respiratory and/or fecal specimens (Table 1).

VirCapSeq-VERT sequencing yielded approximately 320 million single-end reads. Host subtraction removed 88.7% of the reads, resulting in approximately 36 million reads for further analysis. *De novo* assembly yielded approximately 271,000 assembled contiguous sequences (contigs) and 9.7 million unique sequence reads (singletons). NCBI nucleotide BLAST analysis enabled assignment of 10,623 contigs and 4.9 million singletons to viruses. The control CSF specimens containing wild poliovirus type 1 yielded 65 and 29 reads for poliovirus, with genome recovery up to 60.7%. EV-A71 and E-25 (in samples AFM-07 and non-AFM-16, respectively) identified by PCR and Sanger sequencing were also detected by VirCapSeq-VERT. We did not detect reads for viruses other than EV in CSF samples. For samples AFM-07 and non-AFM-16, VirCapSeq-VERT produced 16,994 and 58,206 EV reads, respectively, comprising 99.0% and 74.8% of their genomes (Table 2). Complete viral genomes (GenBank accession no. MK800119 and MK800121) were recovered through gap-filling PCR and amplification of the 5′ and 3′ untranslated regions (UTRs).

**TABLE 2:** Characteristics of enteroviruses recovered from CSF samples using VirCapSeq-VERT

Sample no.	Status	No. of reads		EV genomic length (bp)	Polyprotein bp position (aa length)	Accession no.	% genome coverage	Closest neighbor (accession no.)	% identity		Real-time PCR result (copies/ml)
		Raw	Mapped						nt	aa	
AFM-07	AFM	3,880,269	6,994	7,412	748–7309 (2,194)	MK800119	99.00	EV-A71 (KU641501.1)	99.00	99.50	11,600
AFM-16	Non-AFM	17,812,593	58,206	7,423	744–7328 (2,195)	MK800121	74.80	E-25 (HM031191.1)	86.30	97.90	267

### EV real-time PCR.

Approximately 11,000 and 270 copies of EV RNA were present per ml of cerebrospinal fluid in AFM-07 and non-AFM-016, respectively (Table 2).

### Development of AFM-SeroChip-1 and high-density peptide microarray analyses.

We searched for indirect evidence of CNS infection through antibody surveys of CSF and sera using a programmable peptide microarray based on the Roche Nimblegen platform (6, 7). This design, the AFM-SeroChip-1, comprised ∼160,000 unique 12-mer peptides with 11 overlapping amino acids (aa) that span the capsid proteins (VP1, VP2, VP3, and VP4) of all human EVs (species EV-A, EV-B, EV-C, and EV-D) and the polyprotein of all West Nile viruses (WNVs), using sequences available from the National Center for Biotechnology Information (NCBI) through 31 January 2019 (Table 3; see Table S1 in the supplemental material). The AFM-SeroChip-1 also contains 1,000 random 12-aa peptides to detect nonspecific binding.

**TABLE 3:**
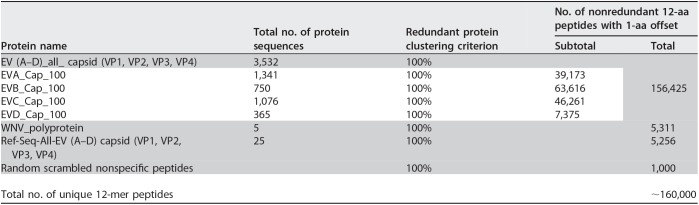
Overview of high-density peptide microarray (AFM-SeroChip-1) peptide components^*a*^

a Shading indicates the total number of peptides from each protein when using the AFM-SeroChip-1 microarray. The white section contains all of the components specifically included in the EV (A-D)_all_capsid data.

10.1128/mBio.01903-19.3 TABLE S1 Enterovirus sequences from GenBank used as input to design the high-density peptide microarray. Download Table S1, XLSX file, 0.1 MB. Copyright © 2019 Mishra et al. 2019 Mishra et al. This content is distributed under the terms of the Creative Commons Attribution 4.0 International license.

Fourteen CSF samples from AFM cases and 27 CSF samples from the 3 control groups (NAC, KDC, and AC) were tested for IgM and IgG antibodies to EV and WNV using AFM-SeroChip-1. Sera were available for testing from only a subset of these 14 subjects (Table 1). CSF samples from the 14 individuals with AFM and 6 members of the NAC group were also tested on arrays that detect antibodies to 8 tick-borne pathogens present in the United States, including Anaplasma phagocytophilum (agent of human granulocytic anaplasmosis), Babesia microti (babesiosis), Borrelia burgdorferi (Lyme disease), Borrelia miyamotoi, Ehrlichia chaffeensis (human monocytic ehrlichiosis), Rickettsia rickettsii (Rocky Mountain spotted fever), Heartland virus, and Powassan virus (7). Antibodies to WNV and tick-borne pathogens were surveyed due to community concerns that these agents might be implicated.

### Identification of immunoreactive conserved peptides in the VP1 capsid proteins of enterovirus (EV-A to EV-D).

**FIG 1:**
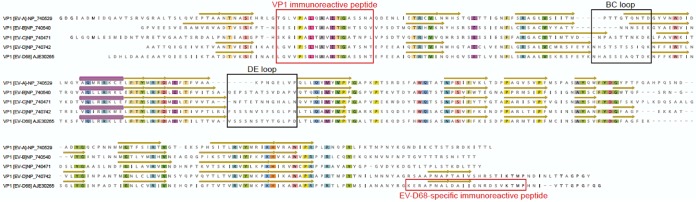
Identification of an immunoreactive peptide sequence region in VP1 protein of reference sequence entries for EV-A, EV-B, EV-C, and EV-D from the National Center for Biotechnology Information (NCBI). VP1 protein models of RCSB Protein Data Bank (RPD) accession no. 4N53, 1COV, E3J48, and 6CSG were used to annotate EV-A, -B, -C, and -D, respectively, for the beta sheets (yellow arrows) and alpha helix (purple tubes). Approximate locations of BC and DE loops are based on analyses by Liu et al. (14) and Imamura et al. (23). Conserved amino acids are highlighted by color. The EV-D68-specific peptide shared less than 70% amino acid identity to other EVs, including EV-D70 and EV-D94.

**FIG 2:**
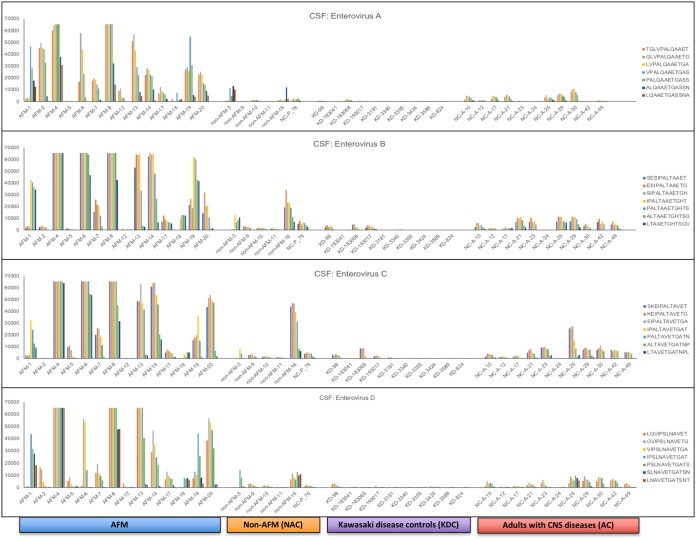
Immunoreactivity against VP1 conserved peptide sequences of EV-A, EV-B, EV-C, and EV-D in cerebrospinal fluid samples of patients with AFM, non-AFM controls (NAC), Kawasaki disease controls (KDC), and adults with CNS diseases (AC). All AFM and NAC specimens were from 2018, except NC-P_76.

High-density peptide microarrays were used to detect immunoreactivity against a comprehensive EV capsid proteome. We identified an 18-aa region (7 overlapping 12-mer peptides from each EV species) (Fig. 1) in the VP1 capsid proteins of EV-A, EV-B, EV-C, and EV-D that was immunoreactive with both serum and CSF from AFM patients (Fig. 2; see Fig. S1 in the supplemental material). This region is highly conserved among EVs and is typically used to infer broad EV immunoreactivity (8, 9). It includes the motif PALXAXETG, with the exception of EV-D, where PSL is substituted for PAL (Fig. 1). CSF samples from 11 of 14 AFM patients (79%) were immunoreactive to this region, compared to those from only 1 of 5 (20%) NAC patients, 0 of 10 (0%) KDC children, and 2 of 11 (18%) AC patients (*P* = 0.023, 0.0001, and 0.0028, respectively).

10.1128/mBio.01903-19.2 FIG S1 Immunoreactivity against EV VP1 conserved peptide sequences of EV-A, EV-B, EV-C, and EV-D in serum samples of patients with AFM, non-AFM controls (NAC), Kawasaki disease controls (KDC), and adult CNS disease controls (AC). Download FIG S1, TIF file, 2.2 MB. Copyright © 2019 Mishra et al. 2019 Mishra et al. This content is distributed under the terms of the Creative Commons Attribution 4.0 International license.

One CSF sample from a member of the NAC group, who had an acute CNS infection with E-25, was also immunoreactive to this conserved EV region **(**Tables 1 and 2). Antibodies to EV peptides were found in CSF from 2 of 11 AC patients (NC-A-25 and NC-A-30) in an intensive care unit with various non-AFM CNS diseases. One of these 2 adults had unspecified epilepsy; the other had multiple sclerosis (Table 1). No CSF samples from the KDC group were immunoreactive with any EV peptide (Fig. 2). None of the AFM or non-AFM patients were antibody positive for West Nile virus or tick-borne disease agents in the CSF. Of the total sera tested, approximately 90% (11/11 [100%] AFM, 2/2 [100%] NAC, 6/8 [75%] KDC, and 5/5 [100%] AC patients) were reactive with one or more EV VP1 peptide sequences (Fig. S1).

### Immunoreactivity against an EV-D68-specific VP1 peptide in AFM patients.

**FIG 3:**
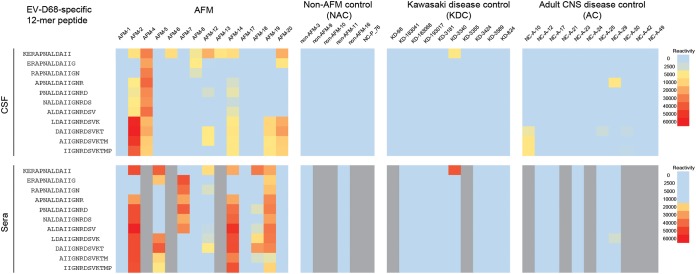
Immunoreactivity against an EV-D68-specific 22-aa VP1 capsid peptide in patients with AFM, non-AFM controls (NAC), Kawasaki disease controls (KDC), and adult CNS disease controls (AC). Respective immunoreactivity intensity measured by the high-density peptide microarrays is shown in heat maps of overlapping 12-mer peptides in the 22-aa EV-D68-specific VP1. Results are shown for cerebrospinal fluid (CSF) in the upper panel and serum in the lower panel. The heat map colors indicate descending reactivity from red, to yellow, to blue. Serum samples not available are indicated in gray. All AFM and NAC specimens were from 2018, except NC-P_76.

Although 70% (18/26) of both AFM and control sera were immunoreactive with at least one VP1 EV-D conserved peptide (Fig. S1), we identified a 22-aa linear peptide sequence in the carboxyl terminus of the EV-D68 VP1 protein that was immunoreactive only in AFM patients (Fig. 1). In AFM patients, this peptide was immunoreactive with 6 of 14 (43%) CSF samples and 8 of 11 (73%) serum samples (Fig. 3). In contrast, this peptide was not immunoreactive with CSF or serum samples from 5 NAC, 10 KDC, or 11 AC patients. Statistical analysis indicated differences between AFM versus NAC, KD, and NC controls for both CSF (*P* = 0.008, 0.0003, and 0.035, respectively) and serum (*P* = 0.139, 0.002, and 0.009, respectively).

## DISCUSSION

Since the summer of 2014, >560 cases of AFM have been confirmed in the United States. The uncertainty underlying the cause for the majority of AFM cases precludes important public health interventions such as development of diagnostics, specific therapy, and preventive measures. Growing evidence suggests that infections with EV, including EV-D68 and EV-A71, may contribute to AFM (4, 10). A potential link to EV-D68 and EV-A71 was proposed based on (i) the presence of viral RNA in some respiratory and stool specimens, (ii) the observation that EV-D68 infection can result in spinal cord infection, necrosis, and paralysis in mice (11, 12), and (iii) the temporal association of respiratory outbreaks of EV-D68 with outbreaks of AFM (4). However, EVs have only rarely been detected in CSF of AFM patients (4 of 567 total confirmed cases studied at the CDC [https://www.cdc.gov/acute-flaccid-myelitis/afm-surveillance.html]).

The potential link to EV, combined with the inability to directly detect its nucleic acids, encouraged us to search for indirect evidence of EV infection in CSF by testing for the presence of EV-specific antibodies. Since EV infection is common in children, traditional immunoassays such as enzyme-linked immunosorbent assay (ELISA) are impeded by cross-reactivity. Virus neutralization assays are considered the “gold standard” but are resource intensive and impractical to implement for >100 types of EV. Accordingly, we employed a microarray system that can simultaneously screen thousands of peptides from all known human EVs with high resolution.

Across the entire EV capsid proteome tiled with 160,000 peptides, the conserved EV VP1 peptides were consistently immunoreactive in CSF of 79% (11/14) of children with AFM, the majority of whom had no EV RNA in CSF. In comparison, NAC patients without evidence of EV RNA in any specimen and KDC patients did not have antibodies to EV peptides in CSF. This conserved peptide region reflects broad EV immunoreactivity, suggesting an association with EV infection and AFM. It is also consistent with EV pathogenesis in mouse models, wherein EVs, including EV-D68, can cause paralytic illness (11, 12).

The demonstration that antibodies to EV peptides are intrathecally produced, such as using an intrathecal ratio of CSF to sera, would provide stronger evidence of CNS infection. We do not have access to sufficient numbers of simultaneously collected CSF and serum specimens to exclude trafficking of EV antibodies from the peripheral circulation into the CNS. However, we consider this unlikely to explain the presence of antibodies to EV peptides in CSF of AFM patients. Of 16 control patients who had antibodies to EV peptides in serum (3 NAC, 8 KDC, and 5 AC patients), only 2 adults and 1 child also had antibodies against EV peptides in CSF. The child with antibodies against EV peptides in CSF had an active CNS infection, with E-25 detected in the same CSF sample. One of the 2 adults had multiple sclerosis, and the other had idiopathic epilepsy. Furthermore, in a study of unexplained encephalitis in Gorakhpur, India, using the same peptide array platform, only 3 of 77 children with antibodies to EV in serum also had antibodies to EV in CSF. Two of these 3 children had diagnoses of EV meningoencephalitis based on symptoms and EV RNA (coxsackievirus or echovirus) in their CSF (N. Mishra and W. I. Lipkin, unpublished data).

We demonstrated immunoreactivity to EV-D68-specific peptides (located within the VP1 C terminus) in CSF from 43% of AFM patients and no immunoreactivity in any of the CSF samples from NAC, KDC, and AC control groups. Based on structural homology, the VP1 C terminus of the EV-D68 strain has been predicted to form neutralization immunogens and conformational epitopes for putative neutralizing antibodies (13, 14). The same EV-D68-specific peptides were immunoreactive in 8 of 11 (73%) sera from AFM patients, compared to none from the three control groups (NAC, KDC, and AC). These findings may appear to be at odds with seroprevalence studies in children and adults in the United States that indicate near 100% infection rates using neutralization assays (13, 15). One potential explanation is that neutralization assays may detect additional nonlinear conformational epitopes, whereas peptide arrays present only linear epitopes. Reactivity from one peptide region may not be comparable to that of the neutralization assay using the whole virion. In work with sera from children exposed to Zika virus, antibodies to the NS2B protein were specific but were not detected at 6 months in 45% of documented infections (6). Understanding why this EV-D68-specific linear peptide is highly reactive in AFM cases but not in controls may shed light on the immune response to EV-D68.

Our serological findings warrant further investigation into the association of AFM with enteroviruses, including EV-D68. Reports of respiratory illness consistent with viral infection typically occurred several days before onset of weakness in the majority of AFM cases. Our inability to detect EV-D68 RNA in the CSF of AFM patients may reflect lack of virus shedding from CNS parenchyma into the intrathecal compartment. Alternatively, CSF specimens may have been collected after virus was cleared from the CNS.

An important limitation of our study is that controls were not optimally matched to the AFM cases with respect to age, year, or season of collection. Although we tried to test for specificity of findings through use of three control groups, prospective studies with appropriately matched controls from the same EV outbreak season will be critical to testing their validity.

### Conclusions.

While other etiologies of AFM continue to be investigated, our study provides further evidence that EV infection may be a factor in AFM. In the absence of direct detection of a pathogen, antibody evidence of pathogen exposure within the CNS can be an important indicator of the underlying cause of disease. Our findings warrant additional investigation, including testing more specimens, developing confirmatory assays, and designing optimally controlled prospective studies. These initial results may provide avenues to further explore how exposure to EV may contribute to AFM as well as the development of diagnostic tools and treatments.

## MATERIALS AND METHODS

### Virologic testing for AFM surveillance.

In 2018, CDC received specimens from 381 patients suspected to have AFM (https://www.cdc.gov/acute-flaccid-myelitis/afm-cases.html). Classification criteria for a confirmed AFM case are acute flaccid limb weakness and magnetic resonance imaging evidence of a predominantly gray matter lesion that spans at least one spinal segment (https://www.cdc.gov/acute-flaccid-myelitis/hcp/case-definition.html) (16). Suspect cases were considered non-cases if they failed to meet the confirmed case definition or had an alternative diagnosis to explain their symptoms. As part of diagnostic testing for AFM surveillance, CDC tested CSF, respiratory, and fecal specimens from suspected AFM cases for EV using a real-time reverse transcription-PCR (rRT-PCR) EV assay (17) and an EV typing assay by VP1 seminested PCR and Sanger sequencing (8). Specimens were also tested for parechoviruses using rRT-PCR (18). Respiratory specimens were further tested using an EV-D68-specific rRT-PCR assay (https://www.fda.gov/medicaldevices/safety/emergencysituations/ucm161496.htm#enterovirus).

### Patients and specimens.

For investigation using VirCapSeq-VERT and peptide microarray approaches, we selected a subset of samples (based largely on available CSF volumes) from AFM cases with onset in 2018, including 13 children and 1 adult. Three groups of controls (Table 1) are included: (i) 5 patients with non-AFM CNS diseases collected during a similar time period as the AFP cases (non-AFM CNS control group [NAC] group; all except one were children), (ii) 10 children with Kawasaki disease (Kawasaki disease control [KDC] group) (19, 20), and (iii) 11 adults with non-AFM CNS diseases (adult control [AC] group). One additional NAC case from 1 child with autoimmune encephalitis (AC-KT-076) during 2019 was also included. NAC and AC controls were included to examine the possibility that antibodies in the peripheral circulation might traffic into the CSF in children and adults with CNS diseases who may have increased permeability of the blood-brain barrier. Paired CSF and serum samples were available for 11 children with AFM, 2 children from the NAC group, 8 children from the KDC group, and 5 adults from the AC group. As an internal positive control for VirCapSeq-VERT performance, we included two RNA extracts from stored CSF specimens of paralytic type 1 wild poliomyelitis cases from Republic of Congo in 2010 (21).

### VirCapSeq-VERT analysis.

The VirCapSeq-VERT capture probe library comprises approximately 2 million oligonucleotides that tile the coding regions of genomes for all viral taxa that contain at least one virus known to infect vertebrates (5). Total nucleic acid was extracted from 240 μl of CSF using the NucliSens easyMAG automated platform (bioMérieux, Boxtel, The Netherlands). Illumina libraries were prepared, pooled, and hybridized with the VirCapSeq-VERT probe set prior to sequencing (Illumina HiSeq 4000) (5, 22). Human genomic and ribosomal sequences were subtracted, and the remaining sequences were analyzed for viral sequences using MegaBlast and BLASTX against the GenBank nonredundant nucleotide and protein databases, respectively.

### Assessment of VirCapSeq-VERT efficiency in detection of enterovirus RNA.

To determine the efficiency of VirCapSeq-VERT in EV genome recovery, we quantitated the amount of EV template in CSF by quantitative reverse transcription-PCR (qRT-PCR). A one-step qRT-PCR assay that targeted a conserved region within the 5′ untranslated region (UTR) was performed on all CSF samples. Ten microliters of total nucleic acid extract was reverse transcribed using Superscript III (Life Technologies). The qRT-PCR assay was performed using 2 μl of cDNA, EV-F primer (TCCTCCGGCCCCTGAATGYGGCTAAT), EV-R primer (GGAAACACGGWCACCCAAAGTA), and EV-probe (6-carboxyfluorescein [FAM]-GCAGCGGAACCGACT-MGB) in a Bio-Rad Touch-CFX 96 real-time PCR instrument at 95°C for 2 min followed by 45 cycles of qPCR at 95°C for 15 s and 60°C for 1 min.

### SeroChip analyses.

Samples were diluted in binding buffer (0.1 M Tris-Cl, 1% alkali soluble casein, 0.05% Tween 20, and water) and hybridized on the AFM array overnight (16 h) at 4°C on the flat surface. After incubation, arrays were washed 3 times for 10 min each on a Little Dipper processor (SciGene; catalog no. 1080-40-1) with 1× TBST (Tris-buffered saline plus 0.05% Tween 20) at room temperature. Secondary antibodies were diluted to 1:5,000 in binding buffer at a concentration of 2 μg/ml. Secondary antibody incubation was done in a plastic Coplin jar (Fisher Scientific; catalog no. S90130) for 3 h at room temperature with gentle shaking on a rocker shaker. First, arrays were incubated with Alexa Fluor 647 AffiniPure goat anti-human IgG that was Fcγ fragment specific (Jackson ImmunoResearch Labs, Inc.; catalog no. 109-605-098), followed by three 10-min washes. Then arrays were incubated with AffiniPure goat anti-human IgM that was Fc5μ fragment specific (Jackson ImmunoResearch Labs, Inc., no. 109-005-043). Incubation with secondary antibody was followed by three 10-min washes with 1× TBST at room temperature and spin-drying. The array slides were scanned on a NimbleGen MS 200 microarray scanner (Roche) at a 2-μm resolution, with excitation wavelengths of 635 and 537 nm simultaneously. In the images recorded with the laser scanner, the relative fluorescent unit (RFU) signals for all the probes were extracted using Roche Sequencing Solutions image extraction software. The RFU signals for all the probes were extracted from the images using Roche Sequencing Solutions image extraction software. The RFU signals were converted into intensity plots after quantile normalization, background and spatial correction, and deconvolution for redundant peptides. An epitope was considered reactive when the signal intensity for at least three contiguous 12-mer peptides was above the threshold. The signal threshold was defined for each reactive epitope by calculating the mean plus 3 standard deviations of the signal intensity for the same epitope in the negative-control samples.

The array data, in the form of relative fluorescence signal intensities from the scanned images (arbitrary units [AU]), were spatially and background corrected and quantile normalized. Signal data points were filtered to retain only immunoreactive peptides that showed the mean +2 standard deviations (>10,000-AU signal for serum and >3,500-AU signal for CSF based on measured values for reactivity against scrambled nonspecific peptide sequences). A region was considered immunoreactive when the signal intensity for at least three contiguous 12-mer peptides was above the threshold. The signal threshold was defined for each immunoreactive epitope by calculating the mean +2 standard deviations of the signal intensity for the same epitope in negative-control samples (6, 7). Immunoreactive peptides were compared with a comprehensive EV protein alignment to scan for type-specific signals.

### Assessment of immunoreactivity signal intensity from the high-density peptide microarray among cases and controls.

For the EV VP1 conserved peptide and EV-D68-specific peptide sequences, comparison of immunoreactivity signal intensity between AFM, non-AFM, Kawasaki disease, and adult CNS groups were evaluated using the Wilcoxon test for paired samples (JMP, v13.0.0; SAS Institute, Inc., Cary, NC).
