# Supplementary material for: Antibodies to Enteroviruses in Cerebrospinal Fluid of Patients with Acute Flaccid Myelitis
Source: mBio. 2019 Aug 13;10(4):e01903-19. doi: 10.1128/mBio.01903-19 (PMC6692520; doi:10.1128/mBio.01903-19)
Supplement: TEXT S1 [file mBio.01903-19-s0001.docx]

**Supplementary Appendix**

**AFM and NAC sample characteristics and comparisons**

A total of 14 AFM patients and 6 non-AFM patients (NAC) were included in this study (**Table 1**); all were from 2018 except 1 non-AFM patient with autoimmune encephalitis from 2019.

|  | Number of Samples | Disease Onset Seasonality (week number) | Age  (years) | CSF Collection Relative to Disease Onset (days) | Sera Collection Relative to Disease Onset (days) |
| --- | --- | --- | --- | --- | --- |
|  |  | medians | | | |
| **AFM** | 14 | 35 | 3 | 2 | 4 (n=11) |
| **Non-AFM*** | 5 | 32 | 11 | 9 | 49 (n=2) |
|  |  |  |  |  |  |
| **P-value (Mann-Whitney)** |  | 0.6317 | 0.0980 | 0.1013 | 0.011 |

**Median ages of KDC, AC, and AFM patients**

Sample collection dates and timing of CSF collections relative to disease onset are not available for comparison.

|  | Number of Sample | Age (years) |
| --- | --- | --- |
|  |  | medians |
| **AFM** | 14 | 3 |
| **KDC** | 10 | 0.5 |
|  |  |  |
| **P-value (Mann-Whitney)** |  | <0.0001 |

|  | Number of Sample | Age (years) |
| --- | --- | --- |
|  |  | medians |
| **AFM** | 14 | 3 |
| **AC** | 11 | 46 |
|  |  |  |
| **P-value (Mann-Whitney)** |  | <0.0001 |
