# Supplementary figures and images for: Antibodies to Enteroviruses in Cerebrospinal Fluid of Patients with Acute Flaccid Myelitis
Source: mBio. 2019 Aug 13;10(4):e01903-19. doi: 10.1128/mBio.01903-19 (PMC6692520; doi:10.1128/mBio.01903-19)

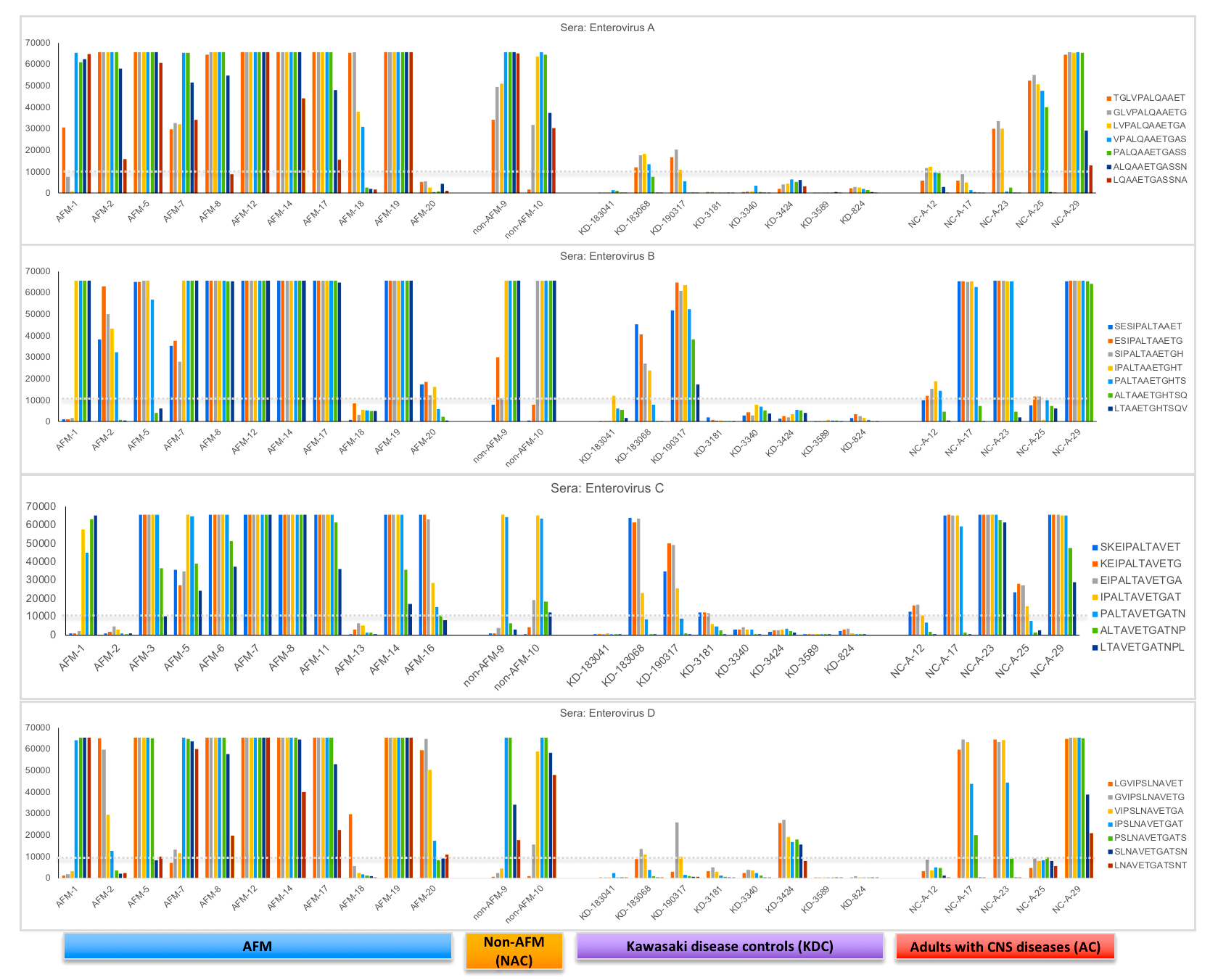

Supplement: FIG S1 [file mBio.01903-19-sf001.tif]
